# Supplementary material for: Measuring the Volatility of the Political agenda in Public Opinion and News Media
Source: Public Opin Q. 2021 Sep 18;85(2):493–516. doi: 10.1093/poq/nfab032 (PMC8530552; doi:10.1093/poq/nfab032)
Supplement: nfab032_Supplementary_Data [file nfab032_supplementary_data.pdf]

Supplementary information for

"Measuring the volatility of the political agenda in public opinion and news media."

Public Opinion Quarterly

Chico Q. Camargo[12], **Peter John**[3], **Helen Z. Margetts**[14], **Scott A. Hale**[14*]

[1] Oxford Internet Institute, University of Oxford, 1 St Giles', Oxford, UK

[2] Department of Computer Science, University of Exeter, Streatham Campus, Exeter, UK

[3] King's College London, Strand, London, UK

[4] Alan Turing Institute, The British Library, London, UK

[*] scott.hale@oii.ox.ac.uk

Contents:

1. Total number of issues in the public opinion polls
2. Topic modelling news articles
3. Null model for the effective number of issues

1. Total number of issues in the public opinion polls

As the opinion polls used in this study also cover issues of extremely low attention, we decided to only consider the top 20 issues with the most attention over the whole timespan of each dataset. We also removed or merged issues introduced after 1990 in the British polls and after 2012 in the German polls, as the introduction of new issues to the poll would cause large shifts in both novelty and the effective number of issues which do not necessarily imply shifts in public opinion.

For instance, in the transition from December 2014 and January 2015, the issue *Race Relations/Immigration/Immigrants* issue in the UK MORI polls changed its name to labelled *Race Relations* while a new issue for *Immigration/Immigrants* was introduced. This change in labelling initially produced a spike in the novelty measures from 2014 to 2015, since there was now a new issue (*Immigration/Immigrants*) in the agenda starting with a high importance, as well as an pre-existing issue (*Race Relations*) that had suddenly lost most of its importance. It is clear that this shift in issue importance was not an effect of a major change in the public agenda within a month, but rather a simple consequence of the polling company re-labelling and re-assigning public responses into two separate categories rather than a single category. With that in consideration, we merged both *Race Relations* and *Immigration/Immigrants* issues in our analysis, considering both as a single *Race Relations/Immigration* issue.

After this cleaning, we obtained a total number of 16 issues after 1990 for the UK polls, which stays stable after 1990, and a total number of issues which stays between 15 and 16 for the Germany polls after 2011. This is shown in Figure 9.

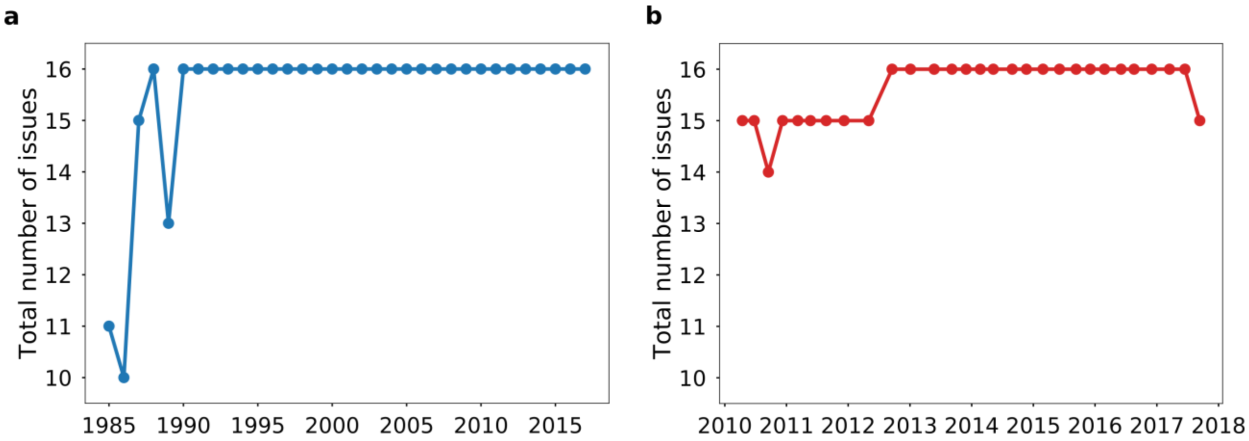

Figure 9. Total number of issues in UK and Germany public opinion polls. Both panels show the change in the number of issues with non-negative attention in the responses in **(a)** the British public opinion survey data and in **(b)** the German opinion polls. Note that the time scales for the data sources differ markedly.

2. Topic modelling news articles

In our analysis, we produced topic models of news media using latent Dirichlet allocation (LDA) to model news articles as distributions over K = 50 topics. Table 1 shows some of these topics for both The Times and Der Spiegel. The topics presented in the table were selected to illustrate the range of topics covered in both news sources. We found almost all topics were interpretable by looking at the top 10–15 words. For example, with words such as "war, defence, army, military, forces" indicating a military-related topic or "moskau, sowjetischen, russen, russischen" indicating a Russia/USSR-related topic.

Source	Topic	Top assigned words	Annotation
The Times	Topic 4	war defence army military forces troops force general british north armed soldiers government	Military, war
The Times	Topic 13	fashion food look like black white good dress wear clothes wine little red buy shop	Fashion
Der Spiegel	Topic 1	patienten ärzte arzt menschen mediziner krankheit professor jahren behandlung leben tod klinik körper krankenhaus blut	Health, medicine
Der Spiegel	Topic 3	moskau sowjetischen russen russischen moskauer sowjet-union sowjetische gorbatschow russische westen sowjets rußland stalin	Russia, USSR

Table 1. Example topics for one run of the topic model with 50 topics.

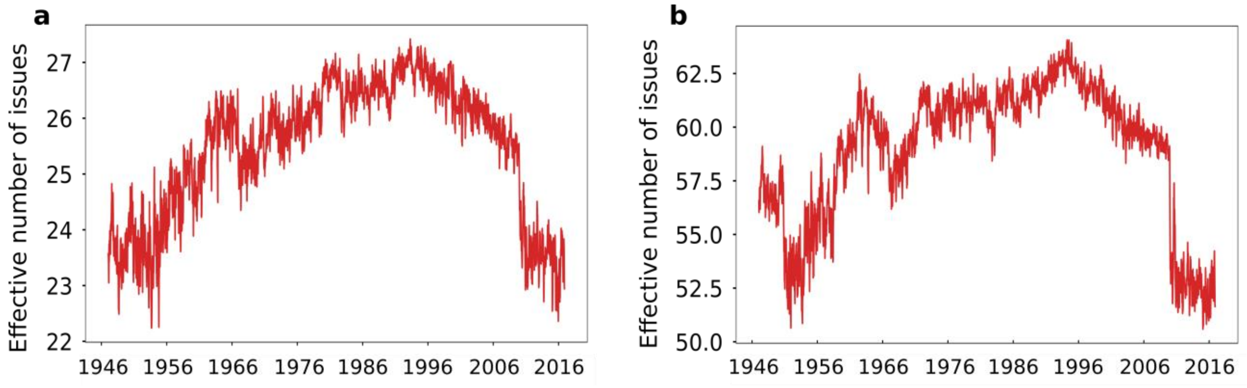

Figure 10. Different choices for the number of topics produce similar results. Time series plots showing the effective number of issues in the distribution of attention to different issues over time, in Der Spiegel, when modelled using **(a)** 30 and **(b)** 70 topics.

We confirm that the choice of *K* topics does not affect our results by modelling The Times and Der Spiegel articles as distributions over number of topics ranging from *K* = 10 to *K* = 90 topics. The corresponding effective number of issues is shown in Figures 10a and 10b for *K* = 30 and *K* = 70 respectively. Note how both plots show similarly shaped curves as in the model with *K* = 50 topics presented in Figure 6. The effective number of issues varies only in absolute value from one panel to another, due to the degree of fine-graining of the topic model, which determines the maximum number of issues. This suggests that the dynamics we observe are not the result of a specific choice of *K* topics. The same is observed for the novelty over time, which is not affected by the choice of *K*, for both news sources.

3. Null model for the effective number of issues

From 2000 to 2010, the effective number of issues in Der Spiegel dropped considerably. This is shown in Figure 6b, for a model with 50 topics and also in Figure 10 for models with 30 and 70 topics. We were initially concerned that this might be due to a reduced number of articles: Der Spiegel output drops from approximately 130 to 60 articles per week around the same time, as shown in Figure 11a.

We address the relationship between the effective number of issues per week and the number of articles per week by defining a null model of an article distribution over K topics. We define the null model as a Dirichlet distribution of order K with parameters $\alpha_1,\ldots,\alpha_K = 1$, that is, picked from an uniform sample over the space of distributions of attention over K topics. For every week with n articles, we then take n uniform samples of this space, i.e., n distributions of attention over K topics, and calculate the effective number of issues for each sample. This produces the curve shown in grey in Figure 11b, which indicates how the effective number of issues of a (uniform) random sample of articles should depend on the number of articles.

For weeks with 130 and 60 articles, the null model predicts the effective number of issues would vary from approximately 46 to 45 issues. In contrast, the empirical data shows a much larger decrease. The effective number of issues in Der Spiegel drops from approximately 43 issues in 2000 (when there were 130 articles per week) to 37 issues in 2010 (where there were 60 articles per week). Not only are both values below the null model prediction, but the difference is notably larger than predicted by the null model. This suggests the drop is not simply due to a reduced number of articles, but rather due to a reduced number of themes covered by the news magazine.

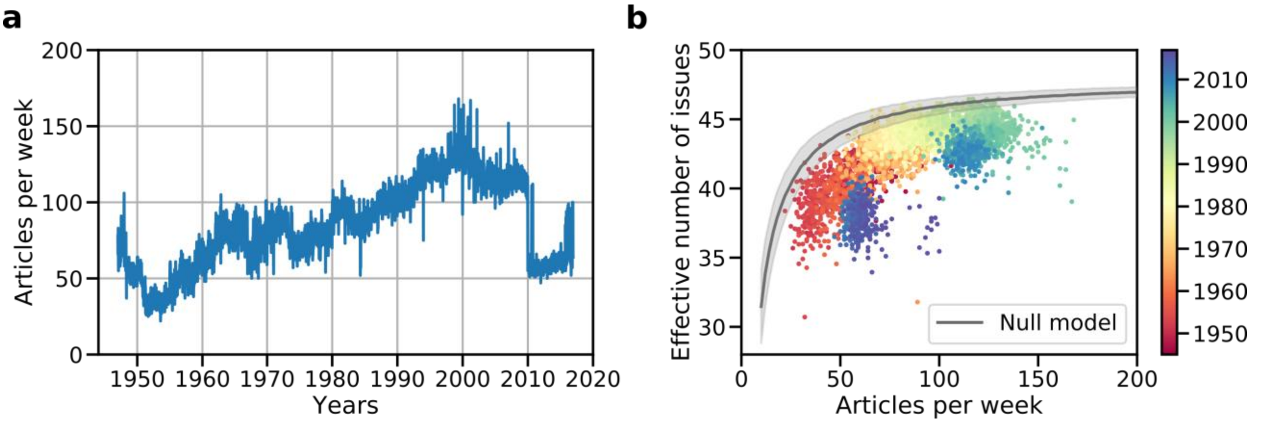

Figure 11. Effective number of issues drops more than expected taking into account the decline in the number of articles published per week. Panel **(a)** shows the number of articles per week for Der Spiegel, which ranges from 40 to 160 articles. From 2000 to 2010, this number drops from approximately 130 to 60 articles per week. Panel **(b)** shows how this number evolves over time, in comparison with the effective number of issues per week. In this panel, the color scale indicates the publication year, while the grey line indicates the null model expectation for the effective number of issues as a function of the number of articles per week. From 2000 to 2010, the null model predicts the effective number of issues should drop from approximately 46 to 45. In contrast, the effective number of issues in Der Spiegel drops from around 43 issues in 2000 to 37 issues in 2010, which suggests the news magazine is covering fewer issues than expected even once controlling for the decrease in the number of articles.
